# To the Editors of the Medical and Physical Journal

**Published:** 1803-03-01

**Authors:** J. Gilbert

**Affiliations:** Derby


### *To the Editors of the Medical and Physical Journal.*

GENTLEMEN,

I Avail myself of the means your excellent publication affords, to communicate to the public some improvement in the treatment of Scarlatina.

The feeble aid afforded by the Peruvian bark in this dangerous disease induced me to try the effect of Digitalis, and of its success I have happily many living proofs to produce.

Scarlatina and Angina Maligna (if they admit of distinction) have been the prevailing complaints in this place for the last eight months. Scarlatina has existed under every degree of violence, and a few cases have been marked with an unusual appearance of malignancy, the patient expiring in thirty-two hours from the period of the attack, while others struggled with the disease, ten or twelve days, and then sunk; the early stage of Scarlatina was here characterized by every symptom of a violently inflammatory affection, and in a few was purely so throughout; its generally rapid change however to a state of extreme debility, renders the successful treatment of it not a little difficult, and tends often to a fatal termination.

I have given the digitalis during every stage of scarlatina, but at no period have I found its effects so evidently salutary, as at that when the patient is sinking and exhausted from the previous violent strong arterial action; the pulse, rapid, feeble, and unequal, gains by its influence that steadiness and strength which marks the return of health, and prevents those anasarcous swellings which often succeed to other modes of treatment. The many facts produced to me during the exhibition of this remedy, leave no doubt on my mind, as to its specific powers at a certain period of scarlatina; and this will justify a hope that its use will become universal, and prove to practitioners a powerful means of effectually opposing the progress of this disease. How far the giving small doses of digitalis would be a security against the contagion of scarlatina, I have not attempted to ascertain; it is a subject, however, well worth investigating.

I remain, &c.

*Derby,*

*January* 29, 1803.

J. GILBERT.

